# Psychological inoculation protects against the social media infodemic

**DOI:** 10.1038/s41598-023-32962-1

**Published:** 2023-04-08

**Authors:** Robert McPhedran, Michael Ratajczak, Max Mawby, Emily King, Yuchen Yang, Natalie Gold

**Affiliations:** 1 Behavioural Practice, Behavioural Practice, Kantar Public UK, 4 Millbank, London, SW1P 3JA UK; 2 Centre for Philosophy of Natural and Social Science (CPNSS), London School of Economics: London School of Economics and Political Science, Houghton Street, London, WC2A 2AE UK; 3 Department of Linguistics and English Language, Lancaster University, Bailrigg, LA1 4YL Lancaster, UK

**Keywords:** Psychology, Human behaviour

## Abstract

Misinformation can have a profound detrimental impact on populations’ wellbeing. In this large UK-based online experiment (n = 2430), we assessed the performance of false tag and inoculation interventions in protecting against different forms of misinformation (‘variants’). While previous experiments have used perception- or intention-based outcome measures, we presented participants with real-life misinformation posts in a social media platform simulation and measured their engagement, a more ecologically valid approach. Our pre-registered mixed-effects models indicated that both interventions reduced engagement with misinformation, but inoculation was most effective. However, random differences analysis revealed that the protection conferred by inoculation differed across posts. Moderation analysis indicated that immunity provided by inoculation is robust to variation in individuals’ cognitive reflection. This study provides novel evidence on the general effectiveness of inoculation interventions over false tags, social media platforms’ current approach. Given inoculation’s effect heterogeneity, a concert of interventions will likely be required for future safeguarding efforts.

## Introduction

In this information age, more than half of humanity can instantaneously retrieve and consume an immense volume of data via the internet^1^. While this access facilitates opportunities for learning, it also provides a platform for the viral transmission of inaccurate and sometimes deleterious information. Such misinformation has the ability to disturb the foundations of our society, undermining citizens’ trust in health treatments and public health measures^2^; creating political unrest^3^; and even destabilising the economy^4^.

Although the true scale of misinformation remains unclear, research suggests its online footprint is expansive. According to estimates from Facebook itself, in April to June 2020, 98 million labels were attached to spurious COVID-19 posts on the platform and 7 million additional posts were removed^5^. Further, a recent study estimated that throughout the COVID-19 pandemic, approximately 2% of web traffic engagement and 14% of Facebook engagement in the US, UK, France, and Germany was with ‘untrustworthy’ news outlets^6^. Despite this large absolute volume, research suggests that misinformation may only comprise a small portion of individuals’ daily media intake. In a study conducted by Allen et al. (2020), the authors determined that ‘fake news’ comprised less than 1% of American’s daily consumption, with most consuming news content via mainstream channels such as television news^7^.

Once present in individuals’ social media feeds, misinformation can be greatly amplified by a small number of users, bots and news outlets^8^: this can occur directly via shares, or indirectly via reactions such as ‘liking’^9^. In fact, there is some evidence to suggest that the diffusion of misinformation is more swift than legitimate information, at least on certain platforms. In one study which analysed the propagation of misinformation on Twitter, the researchers observed that falsehoods spread more rapidly and reached significantly more people than accurate information, particularly in the case of political misinformation^10^. Elsewhere, Cinelli et al. (2020) applied an epidemiological framework to determine the likelihood of a pandemic of false information—an ‘infodemic’—by estimating the reproduction number ($R_{0}$) of misinformation posts on social media platforms^11^. The authors concluded that, while the estimated $R_{0}$ of platforms varied, all were greater than 1, suggesting ‘growth’ and the potential for population spread.

It is thought that the spread of misinformation occurs, at least in part, because humans are notoriously poor at determining the veracity of information^12^. While there are several explanations for this phenomenon, one of the foremost accounts stems from psychological literature on reasoning: specifically, dual-process models of cognition^13^. In dual-process models, immediate automatic, intuitive (‘System 1’ or ‘Type 1’) processes are thought to predominate, unless they are over-ruled by deliberative reflective (‘System 2’ or ‘Type 2’) processes^14^. In the context of online environments such as social media, the volume of content vying for individuals’ attention may compromise their ability to attend to and reflect upon each post^15^. The Cognitive Reflection Tests (CRT)—both the original^16^ and alternate forms^17^—are often used to measure individuals’ ability to over-ride intuitive, incorrect responses to stimuli. Scores on the CRT have been shown to be associated with a variety of cognitive measures, including the heuristics-and-biases tasks^18^, verbal intelligence and numeracy^19^. In addition, CRT performance has been shown to correlate (r = 0.22–0.27) with susceptibility to, and engagement with ‘fake news’^20^. Specifically, those who score lower on the CRT are less able to identify misinformation headlines^20^; and engage in more problematic behaviour on Twitter, such as disseminating unreliable news sources and participating in echo chambers^21^.

Given the volume of misinformation on social media, coupled with the innate fallibility of humans’ information processing, numerous interventions have been applied in an attempt to reduce engagement with misinformation. In many countries, social media platforms self-regulate content, an approach which has been codified in documents such as the European Commission’s Code of Practice on Disinformation^22^. Platforms such as Facebook, Instagram^23^ and TikTok^24^ take a debunking approach to countering misinformation, using fact-checking networks to identify, flag or remove spurious or potentially harmful posts. False tags—sometimes referred to in the literature as ‘content flags’ or ‘flags’^25^—are a choice architecture intervention in which misinformation posts are marked as being disputed by third-party fact checkers^26^. In the false tags currently employed by Facebook, the content of posts is obscured by an interstitial which notifies users of the ‘false information’ and contains two additional buttons: ‘See why’, which contains information on fact-checks; and ‘See post’, which removes the interstitial and allows interaction with the post^27^.

While presently implemented by platforms, the empirical evidence on the efficacy of these interventions is sparse. In a recent online experiment, Kreps and Kriner (2022) examined the impact of Facebook’s current approach to false tags on participants’ perceptions of post accuracy and hypothetical sharing behaviour^5^. The tag did not have a statistically significant impact upon these outcomes, with similar average accuracy assessments and self-reported propensity to share observed in the treatment and control groups. Elsewhere, in another recent online study, ‘rated false’ tags were added to an article headline in one arm; ‘disputed’ tags (Facebook’s original approach) were added to the second intervention arm; and the control remained without tags^25^. ‘Rated false’ tags lowered the participants’ perceptions of posts’ accuracy more than adding a ‘disputed’ tag relative to a control condition; however, the observed effect sizes were small.

In addition to the approaches being implemented by social media platforms, one of the most promising intervention typologies is ‘inoculation’ interventions, which aim to reduce the influence of misinformation ex-ante^28^. Inoculation interventions typically comprise two elements: threat and refutational pre-emption. Threat involves informing individuals that they will see misinformation on social media, while refutational pre-emption—alternatively termed ‘pre-bunking’—involves exposing people to a pre-treatment that builds their resilience to misinformation messages^29^. For example, in *Bad News*, a gamified immunisation intervention encompassing a simulation of Twitter, participants were exposed to examples of six manipulation techniques often used in misinformation, thereby increasing their ability to spot misinformation^30^.

Inoculation interventions have been shown to reduce susceptibility across policy domains, with targeted approaches used to address health^29^, political^31^ and environmental^32^ misinformation. Even more promisingly, in recent years, gamified inoculation interventions have been shown to provide generalised protection against misinformation, including against real-life posts^33^ and content that uses manipulation techniques that differ to those directly addressed in the intervention^34^. Protection conferred by inoculation appears to have an effect that exceeds a month in duration^31^ and remains efficacious when information is presented in a non-gamified video format^35^. Inoculation has also proven effective when operationalised across experimental settings, including in realistic social media simulations^30,33^ and in the field on actual social media platforms such as YouTube^35^. The consistent success of this intervention type has led researchers to consider whether ‘herd immunity’ could be conferred upon a population if enough people were inoculated^36,37^.

While the experiments outlined above provide promising evidence on the potential efficacy of inoculation and false tag interventions, all determine effectiveness using stated intention or perceived accuracy outcomes. This is problematic, as such outcome measures may not be closely associated with real-life reactions to misinformation posts. Critically, the reported associations could be spurious due to experimenter demand effects^38^ or the intention-behaviour gap, in which there is a discrepancy between stated intentions and performed actions^39^. Thus, there is a clear research need for evidence involving an experimental design that uses behavioural, rather than self-reported, outcome measures.

In this study, we investigated the efficacy of inoculation and false tag interventions at reducing engagement with different misinformation ‘variants’, with real-life stimuli sourced directly from social media platforms. In contrast to the extant body of literature, we used a realistic simulated social media platform to directly measure engagement with misinformation, and fitted mixed-effects models to augment the generalisability of our findings. We further investigated whether cognitive reflection (operationalised using the CRT and CRT-2) moderates the efficacy of these interventions.

## Results

### Data summary

**Figure 1:**
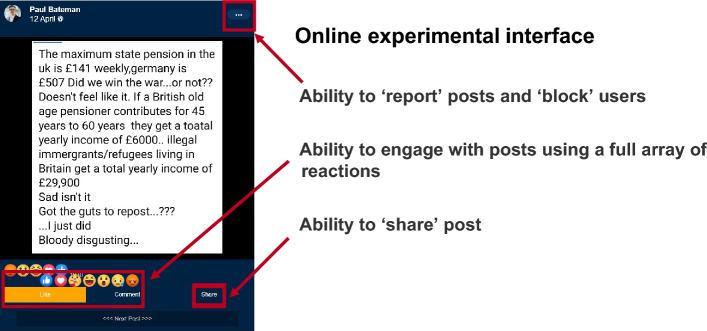
Example screen from the mock social media interface. This proprietary social media simulation was built in Forsta, an online survey platform. Participants could engage with each post as they would on social media, including ‘reacting’ to, ‘commenting on’, ‘sharing’ and ‘reporting’ content. Additional details on the interface can be found in the “Methods” section below.

This study was conducted online, using a realistic mock social media interface. Participants were exposed to 30 posts in a feed—spanning health, politics and finance—15 of which were misinformation and 15 of which were legitimate information. All misinformation posts were genuine live examples sourced from Full Fact, an independent fact-checking organisation, or Reuters, a news agency, both based in the UK^40,41^. Upon presentation, participants could ‘comment’ upon, ‘share’, or ‘report’ posts; or ‘react’ to posts using a full range of emojis. Figure 1 shows a screenshot of the social media interface.

There were three arms in this experiment: an inoculation intervention, a false tag, and a control. The inoculation intervention took the form of a short-form training session which comprised both the ‘threat’ and ‘pre-bunking’ components that are characteristic of this intervention typology^28^. Specifically, participants were initially warned about the danger that misinformation poses to them and were subsequently presented with post attributes that may be used to identify misinformation on social media, loosely based on a source reliability checklist designed by the Modern Language Association^42^. This intervention aimed to provide broad-spectrum defence against misinformation: in focusing on post attributes rather than a particular issue, it intended to confer protection against different forms of manipulation^43^. Participants were then tasked with selecting and ordering the three attributes that are most important to them in establishing the veracity of information.

The false tag intervention was similar to the approach currently used by Facebook: it also applied an interstitial, which notified users of ‘false information’ and contained ‘See why’ and ‘See post’ buttons. Tags were applied to 9/15 misinformation posts, which reflected the proportion of posts tagged in a study that tracked more than 100 COVID-19 misinformation posts over a three-month period^44^. A short video of the false tag intervention—and the general user experience of the social media simulation—can be found in the project’s Github repository, listed in the ‘Availability of data and materials’ section.

This study had three behavioural outcome measures. The study’s primary outcome was positive engagement with posts (clicking the ‘like’ or ‘love’ reactions); the first secondary outcome was any engagement (clicking the ‘like, ‘love, ‘care’, ‘haha’, ‘wow’, ‘sad’, or ‘angry’ reactions); and the second secondary outcome was ‘sharing’ posts (selecting any of the ‘share’ options).

The outcomes were chosen for two reasons. First, ‘liking’ and ‘reacting’ inform social media platforms’ algorithms for content prioritisation in users’ feeds^45,46^; therefore, these behaviours play a crucial role in determining whether misinformation becomes viral or not^47^. Second, for a majority of the posts selected for inclusion in this study, the number of ‘likes’ and ‘reactions’ to posts outnumbered the number of ‘shares’, suggesting that the selection of these actions would reduce the likelihood of problematically zero-inflated outcomes. Nonetheless, ‘sharing’ was retained as a secondary outcome because of its importance in driving post virality.

In addition, the use of behavioural outcome measures—as opposed to stated intention or attitudinal questions—serves a threefold role in this research. First, such outcomes are more ecologically valid than the alternatives: engaging with content in a realistic simulated interface more closely resembles actual use of social media. Second, such outcomes arguably reduce the likelihood of experimenter demand effects: in allowing a large range of potential interactions with each post—and nesting misinformation posts in a simulated ‘feed’ alongside legitimate posts—the experimenters’ expectations are better concealed. Third, this approach is largely novel in the extant literature, and therefore provides unique information about social media behaviour that complements the results of previous research.

### Demographics

We recruited 2653 participants for this study from Prolific, an online access panel. Quota targets on age, sex and ethnicity were enforced to ensure the experiment’s sample broadly resembled the UK population in terms of these demographic characteristics. All participants provided informed consent to participate in the study.

In accordance with our pre-registered approach, following recruitment into the study, participants were screened on the basis of their social media use: only those who had used a social media platform in the preceding year were included in the final sample. Additionally, to ensure that the study contained only participants who were properly engaged, those who failed the study’s attention check question were excluded from the from final sample.

The demographic composition of the final sample of n = 2430 participants can be seen in Table 1.

**Table 1:** Demographic composition of the final sample.

Demographic characteristic	%	n
Gender		
Male	49.1	1194
Female	50.2	1220
Other	0.7	16
Age		
18–24	10.5	256
25–34	20.1	488
35–44	20.1	488
45–54	15.3	371
55–64	22.1	538
65 +	11.9	289
Ethnicity		
White	87.2	2118
Black	2.8	69
Asian	6.7	162
Mixed	1.6	40
Prefer not to say	1.7	41

The average completion time for the study was 19 min (mean = 19.19, SD = 11.57), and participants were paid at a rate of £6 per hour to remunerate them for their time.

### Engagement with misinformation

**Figure 2:**
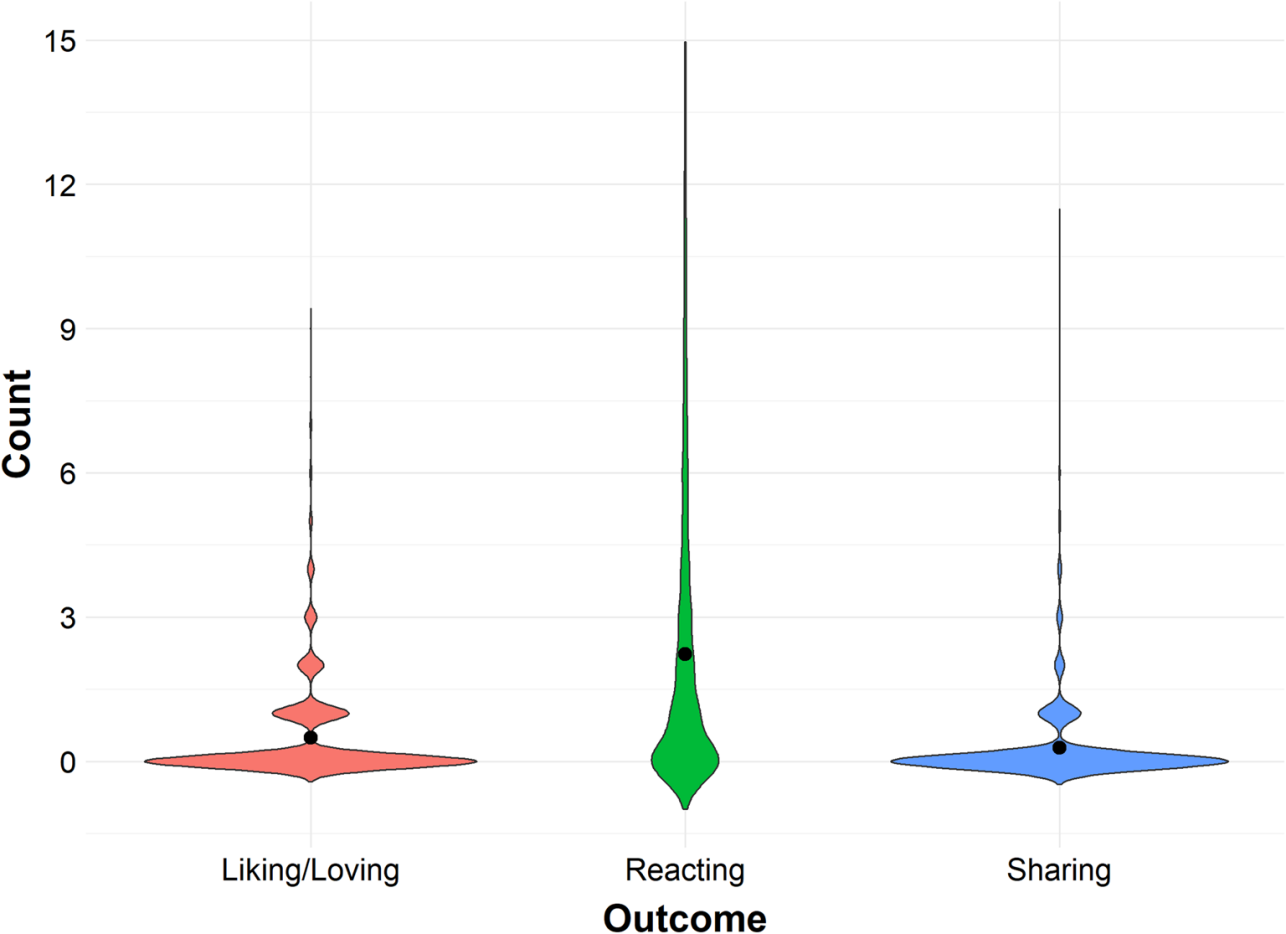
Violin plots of counts by outcome (﻿‘Liking﻿’/﻿‘Loving﻿’, ﻿‘Reacting﻿’, and ﻿‘Sharing﻿’), with average denoted by shaded black circle. The average count of ﻿‘Reacting﻿’ was higher than the average counts of the other two outcomes, whereas the average count of ﻿‘Liking﻿’/﻿‘Loving﻿’ was higher than the average count of ﻿‘Sharing﻿’.

Figure 2 shows the counts of ﻿‘liking﻿’/﻿‘loving’, ﻿‘reacting﻿’, and ﻿‘sharing﻿’ across all misinformation posts. Across the study, positive engagement with misinformation posts was relatively low: 10.9% ‘liked’ or ‘loved’ two or more posts; 16.7% ‘liked’ or ‘loved’ a single post; and 72.3% did not ‘like or’ ‘love’ any misinformation posts. Overall, across stimuli, participants ‘liked’ or ‘loved’ misinformation posts approximately 3.3% of the time.

Total engagement with misinformation was higher: 40.7% reacted to two or more posts; 16.6% ﻿‘reacted﻿’ to a single post; and 42.7% did not ﻿‘react﻿’ to a post. Participants ‘reacted’ to misinformation posts approximately 14.9% of the time.

‘Sharing’ was the most infrequent behaviour. Only 5.4% ‘shared’ two or more posts; 10.6% ‘shared’ a single post; and 84.0% did not ﻿‘share﻿’ a post. Across the experiment, posts were ‘shared’ approximately 1.9% of the time.

### Pre-registered analysis

Our analysis was conducted using five pre-registered logistic mixed effects models (https://osf.io/f3np5). As recommended by Matuschek et al. (2017), we started by attempting to fit models with a maximal specification^48^. Random effects were then removed from these models if they did not improve the goodness-of-fit—as judged using Likelihood Ratio Tests (LRT; α_LRT_ = 0.2)—or if the models did not converge. In using this approach, each model accounted for estimated response variability associated with participants and posts, thereby ensuring a more robust and generalisable estimation of treatment effects^49^.

### Primary and secondary outcomes

As shown in Table 2, the model for the primary outcome (‘liking’ and ‘loving’ posts) indicated that both the false tag (OR 95% CI 0.54–0.84, *p* = 0.001) and the inoculation (OR 95% CI 0.32–0.52, *p* < 0.001) interventions were effective at attenuating positive responses to misinformation. The model’s random intercepts suggest that probability of ‘liking’ and ‘loving’ misinformation posts varied substantially according to participant (*RI*_*Participants*_ = 2.14) and, to a lesser extent, posts (*RI*_*Posts*_ = 0.49).

**Table 2:** Primary and secondary models’ estimates.

Predictors	Liking/Loving			Reacting			Sharing		
	ORs	95% CI	*p*	ORs	95% CI	*p*	ORs	95% CI	*p*
(Intercept)	0.01	0.01–0.02	< 0.001	0.1	0.08–0.13	< 0.001	0.00	0.00–0.00	< 0.001
Arm 2 (False tags)	0.67	0.54–0.84	0.001	0.62	0.49–0.78	< 0.001	0.63	0.36–1.13	0.120
Arm 3 (Inoculation)	0.41	0.32–0.52	< 0.001	0.34	0.25–0.44	< 0.001	0.44	0.23–0.85	0.015
Random effects									
*RI*_*Participants*_	2.14			3.32			23.64		
*RD*_*Arm 2*_				1.10					
*RD*_*Arm 3*_				0.06					
*RI*_*Posts*_	0.49			0.17			0.14		
*RD*_*Arm 2*_				0.03			0.02		
*RD*_*Arm 3*_				0.09			0.19		
ICC	0.44			0.54			0.88		
*N*_*Participants*_	2430			2430			2430		
*N*_*Posts*_	15			15			15		
*N*_*Observations*_	36,450			36,450			36,450		
$R_{M}^{2} /R_{C}^{2}$	0.003/0.07			0.02/0.32			0.003/0.63		

$RI$ refers to random intercepts; $RD$ refers to random differences; ICC refers to intra-class correlation coefficient; $R_{M}^{2}$ refers to marginal $R^{2}$ whereas $R_{C}^{2}$ refers to conditional $R^{2}$ calculated using the delta method^50^.

We examined contrasts to investigate whether interventions performed differentially—both from one another and the control—in suppressing ‘liking’ and ‘loving’. Table 3 indicates that, after adjusting for multiple comparisons using the Bonferroni correction, participants receiving the inoculation intervention were less likely to ‘like’/’love’ misinformation posts than those who received the false tag intervention (OR 95% CI 0.45–0.81, *p* < 0.001).

**Table 3:** Comparisons testing the performance of experimental arms in reducing engagement with misinformation, per model and outcome (p-value adjusted to account for multiple comparisons).

Predictors	ORs	95% Family-Wise CI	Bonferroni Corrected *p*
Liking/Loving			
Arm 1 (Control) vs. Arm 2 (False tag)	0.67	0.51–0.88	0.002
Arm 1 (Control) vs. Arm 3 (Inoculation)	0.41	0.30–0.54	< 0.001
Arm 2 (False tag) vs. Arm 3 (Inoculation)	0.60	0.45–0.81	< 0.001
Reacting			
Arm 1 (Control) vs. Arm 2 (False tag)	0.62	0.47–0.82	< 0.001
Arm 1 (Control) vs. Arm 3 (Inoculation)	0.34	0.24–0.47	< 0.001
Arm 2 (False tag) vs. Arm 3 (Inoculation)	0.54	0.39–0.75	< 0.001
Sharing			
Arm 1 (Control) vs. Arm 2 (False tag)	0.63	0.32–1.26	0.360
Arm 1 (Control) vs. Arm 3 (Inoculation)	0.44	0.20–0.97	0.044
Arm 2 (False tag) vs. Arm 3 (Inoculation)	0.69	0.30–1.59	0.899

To explore the consistency of intervention treatment effects, we ran two additional models which included the study’s secondary outcomes (‘reacting’ and ‘sharing’, see Table 2). For the ‘reacting’ model, we were able to fit a more complex specification, which included random differences—also known as random slopes in the context of numeric variables—with respect to posts and participants. Despite this different specification, the direction and size of observed effects were similar to those observed in the primary outcome model: both the false tag (OR 95% CI 0.49–0.78, *p* < 0.001) and inoculation (OR 95% CI 0.25–0.44, *p* < 0.001) interventions suppressed ‘reactions’ to misinformation relative to the control; further, inoculation was significantly more effective than the false tag intervention in this regard (OR 95% CI 0.39–0.75, *p* < 0.001). (It should be noted that that the estimates of the secondary outcome models were less stable than the estimates of the primary outcome models. Specifically, model convergence was sensitive to the choice of optimiser).

**Figure 3:**
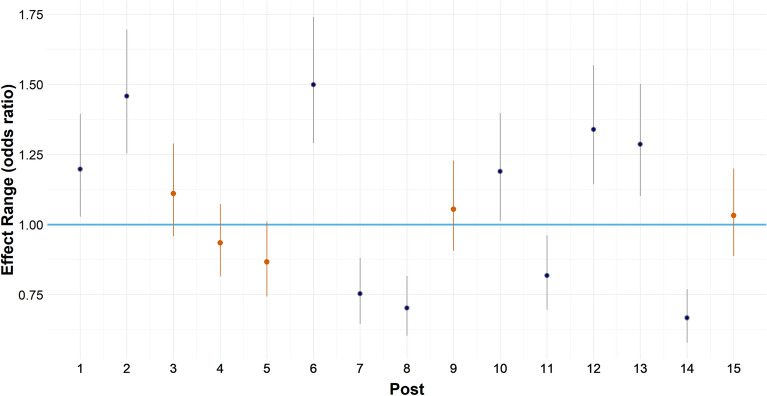
Random differences (Arm 3 | Post), ‘reactions’. Posts varied in their deviations from the global average effect of the inoculation intervention, with some posts having higher odds of being ﻿﻿‘reacted﻿’ to than the average, and other posts having lower odds of being ﻿‘reacted﻿﻿’ to than the average. Posts 1–5 comprised misinformation relating to health; posts 6–10 comprised misinformation relating to politics; and posts 11–15 comprised misinformation relating to finance.

While the analysis above indicates that the inoculation intervention was clearly effective at a total level, the model’s random differences for posts suggested a heterogeneous effect for different forms of misinformation (see Fig. 3). Specifically, for misinformation relating to both politics (posts 6 to 10 in Fig. 3) and finance (posts 11 to 15 in Fig. 3), four of the five estimated effects deviated significantly from the global average. On the other hand, for misinformation relating to health, only two of the five estimated effects deviated significantly from the global average, suggesting less variation in the coverage provided by inoculation for such content.

This result contrasted to the pattern of random differences observed for the false tag intervention (see Fig. S1 in Supplementary Information). For false tags, posts-irrespective of policy domain—did not significantly deviate from the global average.

The inoculation intervention was again effective for ‘sharing’: exposure reduced the odds of ﻿‘sharing﻿’ misinformation posts compared to the control group (OR 95% CI 0.23–0.85, *p* = 0.015). However, in contrast to the other investigated outcomes, the inoculation intervention did not perform significantly better than the false tag intervention (OR 95% CI 0.30–1.59, *p* = 0.899). It should additionally be noted that, given the infrequency with which posts were ‘shared’ in the simulated interface, we were unlikely to have been sufficiently powered for such detailed interrogation of this outcome.

### Moderation models

**Figure 4:**
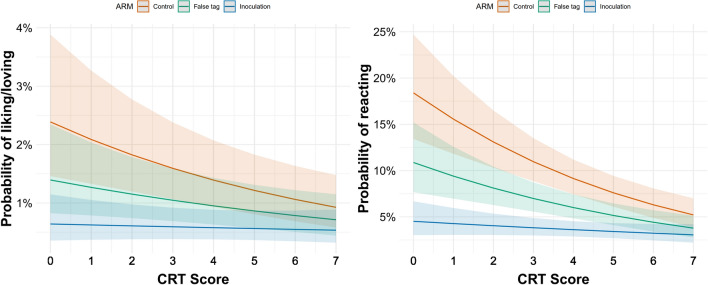
Predicted probably of engaging with misinformation by CRT score and arm. The probability of ‘liking’/loving’ or ‘reacting’ to misinformation posts did not change for participants who received the inoculation intervention, regardless of their CRT score.

In addition to the Primary and Secondary Outcome models outlined above, we ran two moderation models to examine the consistency of the interventions’ treatment effects given differing levels of cognitive reflection. The results of these moderation analyses are visualised in Fig. 4, and further enumerated in Tables S1 and 4.

Table S1 (in Supplementary Information) shows that there was a statistically significant interaction between the inoculation and CRT score for ‘reacting’ (OR 95% CI 1.03–1.29, *p* = 0.011). This interaction can be better understood by further examination of Table 4, which contains estimates of the effects of a one-point increase on the CRT per arm for each outcome.

**Table 4:** The estimated effects of a one-point increase on the CRT, per arm and per outcome.

Predictors	ORs	95% Family-Wise CI	Bonferroni Corrected *p*
Liking/Loving			
Arm 1 (Control) + 1 CRT	0.87	0.79–0.96	0.002
Arm 2 (False tag) + 1 CRT	0.91	0.82–1.00	0.07
Arm 3 (Inoculation) + 1 CRT	0.97	0.87–1.10	1
Reacting			
Arm 1 (Control) + 1 CRT	0.82	0.75–0.89	< 0.001
Arm 2 (False tag) + 1 CRT	0.85	0.78–0.93	< 0.001
Arm 3 (Inoculation) + 1 CRT	0.94	0.85–1.04	0.501

On average, for each one-point increase on the CRT, the odds of ‘reacting’ to misinformation decreased for participants in both the control (OR 95% CI 0.75–0.89, *p* < 0.001) and false tag arms (OR 95% CI 0.78–0.93, *p* < 0.001); however, no such effect was observed for the inoculation arm. A one-point increase on the CRT was also estimated to significantly reduce the odds of participants ‘liking’ or ‘loving’ a misinformation post for participants in the control group (OR 95% CI 0.79–0.96, *p* = 0.002), but not in the false tag and inoculation groups.

### Exploratory analysis

In order to better understand the mechanisms by which the false tag and inoculation interventions work, we ran a series of exploratory models on the study’s legitimate information posts.

The results of this analysis—see Tables S2 and S3, in Supplementary Information—suggested that exposure to the inoculation intervention significantly depressed participants’ odds of ‘liking’/’loving (OR 95% CI 0.61–0.90, *p* = 0.002), reacting to (OR 95% CI 0.47–0.70, *p* < 0.001), and ‘sharing’ (OR 95% CI 0.39–0.68, *p* < 0.001) legitimate posts, compared to the control. On the other hand, estimates suggested that exposure to the false tag intervention did not reduce the odds of ‘liking’, ‘reacting’ to or ‘sharing’ legitimate posts.

## Discussion

In recent years, there has been an abundance of empirical research investigating the effects of different interventions addressing individuals’ susceptibility to misinformation. This online experiment extended this literature by demonstrating the efficacy of inoculation and false tag interventions in shielding against different misinformation variants. It employed ‘live’ fake news posts embedded in a naturalistic social media platform simulation and is the first study to use behavioural responses as outcomes. Further, we fitted mixed-effects models, thereby augmenting the generalisability of our estimated effects to a wider pool of UK residents and misinformation posts^51^.

This study yielded several results of theoretical and practical consequence to social media platforms, researchers and policymakers alike. First, we showed that, while both the inoculation and false tag interventions worked to attenuate engagement with misinformation, inoculation was more effective in this regard. Those in the inoculation intervention arm ‘liked’ or ‘loved’ misinformation at half the rate of those in the control (a 50.5% reduction in the observed behaviour); and reacted to misinformation posts substantially less than the control (a 42.0% reduction in the observed behaviour). This pattern of results accords with a rapidly increasing volume of research that highlights the effectiveness of inoculation interventions. However, in contrast to this existing body of evidence, we noted heterogeneity in inoculation’s protection against different stimuli: random differences analysis suggested variation in effect according to individual post. This finding reinforces two primary challenges associated with reaching population-level psychological ‘herd immunity’: while inoculation interventions that highlight strategies commonly employed in misinformation may provide broad-based immunity^28^, strength of protection inevitably varies depending on both subject matter and the manipulation strategies employed in different posts. Further, the immunity conferred by such interventions is inherently limited to the manipulation techniques used in current misinformation examples. As misinformation mutates into variants that weaponise different persuasive strategies—for example, the deployment of deepfakes—new forms of protection will need to be developed to keep pace^52^.

Second, there were two findings in this study that provided additional evidence about the mechanism by which inoculation interventions works. The results of the moderation analysis suggested that cognitive reflection moderates the effects of the false tag intervention but does not moderate the immunity provided by inoculation, with the false tag intervention being more effective for those with higher CRT scores. This finding is congruent with the results of Roozenbeek, van der Linden, et al. (2022), who found that the effect of inoculation—albeit delivered in video form—was robust to differing levels of cognitive reflection, as measured by the CRT^35^. Further, of consequence to policymakers and regulators, this result provides early evidence that while widely applied by social media platforms, false tag interventions are less effective for the very individuals who are most susceptible to misinformation.

In addition to suppressing engagement with misinformation, the inoculation intervention reduced engagement with legitimate posts, both in terms of ‘reactions’ and ‘shares’. These effects were asymmetrical, with a larger suppression effect estimated for misinformation than for legitimate information, suggesting some improvement in ability to discriminate among inoculated participants. To illustrate this point, participants in the inoculation arm ‘liked’ or ‘loved’ legitimate information slightly less than the control (a 15.0% reduction in the observed behaviour), while a larger effect was observed for ‘reactions’ (a 20.8% reduction in the observed behaviour). Future experiments should aim to further explore the mechanisms by which inoculation works, as there is recent evidence that some inoculation interventions may catalyse more conservative engagement with posts rather than substantially improving discernment^53^. If this is indeed the case, social media platforms, policymakers and regulators will be forced to weigh the benefits of decreasing the spread of misinformation via inoculation against the social costs associated with a reduction in the dissemination of legitimate information.

Third, the false tag intervention—a strategy akin to the approach currently employed by Meta—was effective at reducing engagement with misinformation posts (a 25.0% reduction for ‘liking’/’loving’ and a 20.7% reduction for ‘reacting’), but it did not reduce the odds of engaging with legitimate content. Additionally, the false tag intervention did not significantly reduce the odds of ‘sharing’ misinformation posts relative to the control. This result is particularly worrying given that this behaviour can directly promulgate harmful content including misinformation, and feeds into social media’s algorithms for content prioritisation^45^.

While the former result is positive for social media platforms—as their profitability is inextricably linked with engagement with content^54^—and the latter result is negative, it must be acknowledged that the true effect of this intervention is likely to be different to the one reported in this study. While we randomly applied tags to 60% of the misinformation posts in the mock feed, the proportion of misinformation that is tagged by platforms is unclear. Further, it is reasonable to assume that the overall effectiveness of the false tag intervention varies depending on the proportion of misinformation posts that are tagged as misinformation in the real-world. Thus, we caveat the estimated effect of the false tag intervention in this study and note that its real-world effect would likely be weaker if a smaller proportion of misinformation posts were tagged by content moderators, or stronger if a higher proportion of misinformation posts were tagged.

Fourth, this study demonstrates that realistic simulations of social media platforms can act as invaluable testbeds for misinformation interventions in future years. In contrast to much of the current literature, our study used behavioural outcome measures, ‘live’ misinformation sourced from social media and a naturalistic social media simulation. In doing so, we enhanced the ecological validity of this online study by closely replicating the moments of choice in which citizens encounter misinformation in the wild, considered by some to be a precondition necessary for experiments’ external validity^55^. When considered alongside our use of mixed-effects models—which included random effects for individuals and posts—we contend that the results of this study are more generalisable than those in much of the extant literature.

This study is not without limitations. As noted, the proportion of misinformation posts flagged in the false tag intervention—while based on previous research—may be higher (or lower) than the proportion flagged by social media platforms. Second, we used an online access panel for our sample. Although such sources enable researchers to access a large pool of participants in a relatively short period of time, online access panels notoriously suffer from selection and, to a lesser extent, coverage biases^56^. When biased sources are used to sample a large group of participants, those biases are amplified, thereby manifesting a ‘big data paradox’^57^. The extent to which these biases afflict the sample source used in this study is unclear; therefore, the estimates of effect size should be read with this potential shortcoming in mind.

In spite of these weaknesses, this experiment provides important new evidence on the efficacy of two misinformation interventions frequently discussed in the recent literature. We found that inoculation, in particular, was effective in attenuating engagement; however, the strength of immunity it confers differed for misinformation variants. Given this constraint, both inoculation and false tags may play a role in future safeguarding efforts: as misinformation mutates, a concert of interventions may be required to maximise psychological antibodies.

## Methods

### Participants

We recruited 2653 participants using Prolific, an online access panel. In recruitment, we used parallel quota targets based on the 2011 Census to control for distribution of age, sex and ethnicity. Age was stratified using five brackets: 18–27, 28–37, 38–47, 48–57, and 58 + . Sex was stratified into male and female. Ethnicity was stratified into five categories: White, Mixed, Asian, Black, and Other. Data collection took place from the end of August 2022 to mid-September 2022.

Two primary attention checks were used to identify and remove ‘low quality’ participants. First, any respondent who completed the study three or more standard deviations below the mean completion time was removed from the study. Second, any participants who failed to correctly answer our attention check question was excluded from the study. The attention check took the following form: “For quality control purposes, please select ‘Slightly disagree’ here.” The response options were: ﻿‘Strongly disagree; Disagree; Slightly disagree; Neither agree nor disagree; Slightly agree; Agree; Strongly agree﻿﻿’.

Lastly, in order to ensure that all participants could engage with the full range of misinformation posts in the mock feed, audio and video checks were used to screen out those who were unable to access multimedia preceding the experiment.

After exclusions, we were left with 2430 participants. These participants’ data were used in the main analysis.

### Procedure

Participants were first presented with an introduction screen thanking them for taking part in the study and outlining what participation in the study involved. The introduction screen contained a disclaimer about the inclusion of potentially harmful content that read “Some of the posts you will see may be considered ‘misinformation’. If you do not wish to proceed, please opt out below.”. An opt-out button was provided at this point.

Participants were screened for social media use by asking which of the common social media platforms (including, but not limited to Facebook, Twitter, Snapchat, Instagram, TikTok) they have used within the past 12 months. Potential participants were screened out if they answered: “I haven’t used any social media platforms in the past 12 months”. After passing the social media use screener, participants were randomised to one of three intervention arms, described below.

In Arm 1 (the control), participants were exposed to 15 misinformation posts and 15 neutral posts presented in a random order. In this arm, participants saw a generic version of a social media interface, that they were able to interact with by reacting (﻿﻿‘like﻿’, ﻿‘love﻿’, ﻿‘care﻿’, ﻿‘haha﻿’, ﻿‘wow﻿’, ﻿‘sad﻿’, or ﻿‘angry﻿’ reactions), commenting, sharing, or reporting.

In Arm 2 (false tags), participants were exposed to the same 15 misinformation posts and 15 neutral posts as the participants in Arm 1, but 9 out of 15 misinformation posts were randomly allocated a warning similar to Facebook’s default.

In Arm 3 (inoculation), participants were exposed to the same 15 misinformation posts and 15 neutral posts as the participants in the control arm but, prior to the training screen, they were exposed to a checklist highlighting features of misinformation that define it as misinformation, with pictorial examples. This checklist contained inoculation features identified by Traberg et al. (2022) that characterise it as an inoculation intervention^28^. The full checklist was as follows:

1. Check the source
  Can I see who or where this has come from? ​
  Do I recognise the person or organisation posting? Can I trust them?​
  Does the person posting have expertise, training, or a job that is relevant to what they are posting about?
2. Check the purpose​
  Is this post trying to persuade me to think a certain way?​
  Is the post trying to sell a product or organisation?​
  Is the post a joke or satire? ​
  Is this trying to grab my attention rather than give me facts?​
3. Check the accuracy ​
  Should I consider some other views? ​
  Can I see any statistics or research to back this up? ​
  Have I heard something that says the opposite to this from another source?
4. Check the relevance ​
  Can I see when this was posted?​
  Can I find something more recent on the same topic?​
  Has the content received a positive response?
5. Check the format ​
  Can I see lots of spelling mistakes? ​
  Does the logo or branding look odd to me?​
  Do I think the images are good quality?​ Do I think the visuals could have been altered in some way?

Users viewed the checklist prior to browsing the ‘feed’ and were unable to navigate back to view it more than once.

The simulated social media interface user experience was similar to that of Facebook, but with some differences that should be noted. First, posts were shown on individual screens, and users were required to click ‘Next Post’ to browse further. Second, users could not return to posts once they had clicked ‘Next Post’, meaning their reactions—or non-reactions—to posts were final.

Participants were asked to complete the CRT^16^ and CRT-2^17^ to assess their cognitive reflection. After interacting with 30 posts, participants were asked a series of attitudinal questions relating to social media use.

### Posts

All participants were exposed to 30 social media posts (15 were legitimate and the rest were misinformation). The posts fell into one of three topic categories: health, politics, or finance.

Misinformation posts were sourced from Full Fact, an independent fact-checking organisation^40^ or Reuters, a news agency^41^; both were based in the UK at the time of writing. Posts were selected to include a spread of formats (post with image, meme, text only, or video). Legitimate posts were chosen to balance as closely as possible with the format and topic of their corresponding misinformation post. Some posts were reformatted to adhere to the dimensions of our simulated social media platform.

The 15 misinformation posts were:

Post 1, health misinformation: a meme-based post implying that monkeypox is really shingles induced by COVID-19 vaccination;

Post 2, health misinformation: a video suggesting that monkeypox is an agent of biological warfare;

Post 3, health misinformation: a post containing an image of a text excerpt reporting on a study that claims ‘long’ COVID-19 does not exist;

Post 4, health misinformation: a video suggesting that applying hydrogen peroxide to the skin treats cancer;

Post 5, health misinformation: a post including text and an image which claims that cannabis oil can cure cancer;

Post 6, political misinformation: a post from the Labour party which incorrectly claims a 95% reduction in new affordable homes to buy under the Conservative government;

Post 7, political misinformation: an image-based post containing a fabricated quote on immigration from Labour MP Diane Abbott;

Post 8, political misinformation: a post containing a doctored image of Conservative MP Penny Mordaunt wearing military medals;

Post 9, political misinformation: a text and image-based post containing a fabricated quote from Donald Trump praising Karl Marx;

Post 10, political misinformation: a post containing a doctored image of women protesting against the anti-abortion movement;

Post 11, financial misinformation: a text-only post incorrectly comparing state pension figures and amounts paid to illegal immigrants in the UK;

Post 12, financial misinformation: an image of a recent scam that claims Amazon has created its own ‘AMZ Token’ cryptocurrency;

Post 13, financial misinformation: a post containing a screenshot of a fake report that claims Martin Lewis has endorsed a new cryptocurrency investment platform;

Post 14, financial misinformation: a text-only post providing an inaccurate account of an individual being forced to house refugees in his home;

Post 15, financial misinformation: a post containing both text and image which gives misleading and inaccurate advice on how to save money on petrol.

The 15 legitimate posts were:

Post 16, legitimate health information: a post from the World Health Organisation containing text, an image and information about the side-effects of COVID-19 vaccination;

Post 17, legitimate health information: a video with accompanying text providing information about the Monkeypox virus;

Post 18, legitimate health information: a text-only post from the Office for National Statistics (ONS) providing statistics about ‘long’ COVID-19;

Post 19, legitimate health information: a video and accompanying text containing statistics about cervical cancer;

Post 20, legitimate health information: an image and accompanying text containing a balanced account of whether a meat-free diet can prevent certain cancers;

Post 21, legitimate political information: a post with both text and an image from the housing charity Shelter containing official homelessness statistics;

Post 22, legitimate political information: a post from the UK Home Office outlining a new agreement between the UK and Nigeria to deter illegal immigration;

Post 23, legitimate political information: a post containing a video compilation of speeches made by Prime Ministerial candidate Liz Truss, with accompanying text;

Post 24, legitimate political information: a satirical cartoon accompanied by text outlining the news of UK Prime Minister Boris Johnson’s flat renovation expenses;

Post 25, legitimate political information: a post containing text and an image detailing the Saatchi and Saatchi campaign recreated in response to the recent Roe vs. Wade ruling in the United States;

Post 26, legitimate financial information: a text-based post outlining upcoming rises in the state pension;

Post 27, legitimate financial information: a post containing text and an image highlighting that the bitcoin symbol has been added to the list of currencies supported by Microsoft Excel;

Post 28, legitimate financial information: an image and accompanying text providing a news update on hackers stealing cryptocurrency from bitcoin ATMs;

Post 29, legitimate financial information: a post from HM Government containing text and an image, with information about how those receiving Pension Credit can get paying Council Tax bills; and

Post 30, legitimate financial information: a post containing text and an image providing a list of tips to reduce costs in the cost-of-living crisis.

### Measurement

The primary outcome variable was a binary variable: whether a participant decided to like (1) or not like (0) each misinformation post. In this context, liking was operationalised as a ‘like’ or ‘love’ reaction.

Secondary outcome variables were also binary variables. The first outcome was whether a participant decided to react (1) or not react (0) to each misinformation post (operationalised as a ‘like’, ‘love’, ‘care’, ‘haha’, ‘wow’, ‘sad’, or ‘angry’ reaction). The second outcome was whether a participant decided to ‘share’ (1) or not ‘share’ (0) each misinformation post, operationalised as pressing the ‘share’ button or not pressing the ‘share’ button.

### Statistical power

To run power simulations for logistic mixed-effects models, assumptions about the variance parameters of the random effects were required. In the context of our study, the estimates to be specified concerned the variation in the probability of liking between participants and variation in the probability of liking between misinformation posts. (Note that we omitted the variances associated with random differences, because we did not have reliable estimates of these variances).

We assumed that the effect size of the interventions was similar to those in an unpublished online experiment conducted for the UK Government (the results of which are unable to be shared). This unpublished work was conducted using a similar social media interface as the present study, also involved the presentation of real-life misinformation to participants, and tested relatively similar debiasing and choice architecture interventions.

The effect sizes in our power simulation (drawn from this unpublished study) were: − 2.008 in log-odds for Arm 1 ($\beta_{0}$); − 1.057 in log-odds for Arm 2 ($\beta_{1}$) and − 0.702 in log-odds for Arm 3 ($\beta_{2}$). Consequently, the Arm 1 (at $\beta_{0}$) probability of liking misinformation video was 11.84%. The absolute reduction in the probability of liking in arm 2 ($\beta_{1}$), versus this baseline, was 7.38% (the probability of liking in Arm 2 was 4.46%). The reduction in the probability of liking in in Arm 3 ($\beta_{2}$), versus the same baseline, was 5.60% (the probability of liking in Arm 3 was 6.24%). The absolute difference in the probability of liking a misinformation post between Arm 1 and Arm 2 was 1.78%.

Table S4 (see Supplementary Information) shows the estimates of power under different model assumptions, adjusting for multiple comparisons (using Bonferroni correction), given 1000 simulations per sample size. As can be seen, given our sample size of n = 2400 and effects similar to those above, we were sufficiently powered for all comparisons between the experimental arms (assuming α = 0.05 and requiring a minimum 1-β of 0.8).

### Ethics declaration

All experimental protocols were approved by the LSE Research Ethics Committee. All experiments were performed in accordance with relevant named guidelines and regulations. Informed consent was obtained from all participants.

## Supplementary Information

Supplementary Information.

## Data Availability

The datasets generated and/or analysed during the current study are available in the Github repository or PsyArxiv, https://github.com/MichaelRatajczak92/Misinformation_Project or https://psyarxiv.com/bd2zu/. All data generated or analysed during this study are included in this published article [and its supplementary information files]. Additional detail on the social media simulation and reasonable requests for access may be directed to the corresponding author.
