# Supplementary material for: Psychological inoculation protects against the social media infodemic
Source: Sci Rep. 2023 Apr 8;13:5780. doi: 10.1038/s41598-023-32962-1 (PMC10082776; doi:10.1038/s41598-023-32962-1)

**Supplementary Information**

| **Table S1. Moderation model estimates.** | | | | | | | | | | | |
| --- | --- | --- | --- | --- | --- | --- | --- | --- | --- | --- | --- |
|  | **Liking/Loving** | | | | | | **Reacting** | | | | |
| Predictors | ORs | | 95% CI | | *p* | | ORs | 95% CI | | *p* | |
| (Intercept) | 0.02 | | 0.01 – 0.04 | | <0.001 | | 0.23 | 0.16 – 0.33 | | <0.001 | |
| Arm 2  (False tags) | 0.58 | | 0.35 – 0.96 | | 0.032 | | 0.54 | 0.34 – 0.87 | | 0.012 | |
| Arm 3  (Inoculation) | 0.26 | | 0.15 – 0.46 | | <0.001 | | 0.21 | 0.13 – 0.35 | | <0.001 | |
| CRT | 0.87 | | 0.81 – 0.94 | | 0.001 | | 0.82 | 0.76 – 0.88 | | <0.001 | |
| Arm 2 (False tags): CRT | 1.04 | | 0.93 – 1.17 | | 0.483 | | 1.04 | 0.94 – 1.16 | | 0.464 | |
| Arm 3 Inoculation): CRT | 1.12 | | 0.99 – 1.27 | | 0.079 | | 1.15 | 1.03 – 1.29 | | 0.011 | |
|  |  | |  | |  | |  |  | |  | |
| Random Effects | |  | | |  |  | | |  | |  |
| ${RI}_{Participants}$ | 2.08 | |  | |  | | 3.53 |  | |  | |
| ${RI}_{Post}$ | 0.5 | |  |  | | | 0.11 |  | |  | |
|  |  | |  |  | | |  |  | |  | |
| ICC | 0.44 | |  |  | | | 0.53 |  | |  | |
| $N_{Participants}$ | 2430 | |  |  | | | 2430 |  | |  | |
| $N_{Posts}$ | 15 | |  |  | | | 15 |  | |  | |
| $N_{Observations}$ | 36450 | |  |  | | | 36450 |  | |  | |
| $R_{M}^{2}/R_{C}^{2}$ | 0.004 / 0.07 | |  |  | | | 0.02 / 0.33 |  | |  | |

| **Table S2. Primary and secondary models’ estimates (legitimate posts).** | | | | | | | | | | | | | | | | | | | |
| --- | --- | --- | --- | --- | --- | --- | --- | --- | --- | --- | --- | --- | --- | --- | --- | --- | --- | --- | --- |
|  | **Liking/Loving** | | | | | | |  | | **Reacting** | |  | |  | | **Sharing** | |  |  |
| Predictors | ORs | | | CI | | p | | ORs | | CI | | p | | ORs | | CI | | p |  |
| (Intercept) | | | 0.04 | 0.02 – 0.06 | | <0.001 | | 0.18 | | 0.13 – 0.25 | | <0.001 | | 0.01 | | 0.01 – 0.02 | | <0.001 |  |
| Arm 2  (False tags) | | | 0.94 | 0.77 – 1.13 | | 0.49 | | 0.87 | | 0.72 – 1.06 | | 0.169 | | 0.87 | | 0.67 – 1.13 | | 0.286 |  |
| Arm 3  (Inoculation) | | | 0.74 | 0.61 – 0.90 | | 0.002 | | 0.57 | | 0.47 – 0.70 | | <0.001 | | 0.52 | | 0.39 – 0.68 | | <0.001 |  |
|  |  | | |  | |  | |  | |  | |  | |  | |  | |  |  |
| Random Effects | |  | | |  | |  | |  | |  | |  | |  | |  | |  |
| ${RI}_{Participants}$ | | | 2.58 |  | |  | | 3.25 | |  | |  | | 3.9 | |  | |  |  |
| ${RI}_{Post}$ | | | 0.79 |  | |  | | 0.37 | |  | |  | | 0.37 | |  | |  |  |
|  | | |  |  | |  | |  | |  | |  | |  | |  | |  |  |
| ICC | | | 0.51 |  | |  | | 0.52 | |  | |  | | 0.56 | |  | |  |  |
| $N_{Participants}$ | | | 2430 |  | |  | | 2430 | |  | |  | | 2430 | |  | |  |  |
| $N_{Posts}$ | | | 15 |  | |  | | 15 | |  | |  | | 15 | |  | |  |  |
| $N_{Observations}$ | 36450 | | |  | |  | | 36450 | |  | |  | | 36450 | |  | |  |  |
| $R_{M}^{2}/R_{C}^{2}$ | 0.001 /0.22 | | |  | |  | | 0.01/0.39 | |  | |  | | 0.003/0.17 | |  | |  |  |
| Note: $RI$ refers to random intercepts; ICC refers to intra-class correlation coefficient; $R_{M}^{2}$ refers to marginal $R^{2}$ whereas $R_{C}^{2}$ refers to conditional $R^{2}$ calculated using the delta method (Nakagawa et al., 2017). | | | | | | | | | | | | | | | | | | | |

| **Table S3. Multiple comparisons, per model (legitimate posts).** | | | | | |
| --- | --- | --- | --- | --- | --- |
| Predictors | OR | | 95% Family-Wise CI | | Bonferroni Corrected *p* |
| **Liking/Loving** | |  | |  | |
| Arm 1 vs. 2 | 0.94 | | 0.75 – 1.17 | | 1 |
| Arm 1 vs. 3 | 0.74 | | 0.59 – 0.93 | | 0.006 |
| Arm 2 vs. 3 | 0.79 | | 0.63 – 0.99 | | 0.049 |
|  |  | |  | |  |
| **Reacting** |  | |  | |  |
| Arm 1 vs. 2 | 0.87 | | 0.69 – 1.1 | | 0.508 |
| Arm 1 vs. 3 | 0.57 | | 0.45 – 0.73 | | <0.001 |
| Arm 2 vs. 3 | 0.66 | | 0.52 – 0.83 | | <0.001 |
|  |  | |  | |  |
| **Sharing** |  | |  | |  |
| Arm 1 vs. 2 | 0.87 | | 0.63 – 1.19 | | 0.859 |
| Arm 1 vs. 3 | 0.52 | | 0.37 – 0.72 | | <0.001 |
| Arm 2 vs. 3 | 0.60 | | 0.43 – 0.83 | | <0.001 |

| **Table S4. Power to detect an effect of specified size, by total sample size (N).** | | | | | | | | |
| --- | --- | --- | --- | --- | --- | --- | --- | --- |
| N | $\beta_{1}$ | $\beta_{2}$ | $\gamma_{0j}$ | $u_{0l}$ | $\varepsilon_{ijl}$ | $\beta_{1}-\beta_{0}$ | $\beta_{2}-\beta_{0}$ | $\beta_{2}-\beta_{1}$ |
| 1500 | -1.057 | -0.702 | 1.757 | 0.623 | 0.1 | 100% | 100% | 67.5% |
| 1800 | -1.057 | -0.702 | 1.757 | 0.623 | 0.1 | 100% | 100% | 76% |
| 2100 | -1.057 | -0.702 | 1.757 | 0.623 | 0.1 | 100% | 100% | 82.8% |
| 2400 | -1.057 | -0.702 | 1.757 | 0.623 | 0.1 | 100% | 100% | 87% |

In Table S4, $\beta_{0}$ is the predicted value for a baseline category (Arm 1), whereas $\beta_{1}$ and $\beta_{2}$ represent deviations in the log-odds of liking misinformation posts of Arms 2 and 3, respectively, from Arm 1. $\gamma_{0j}$ is the variance at the participant level $j$, $\gamma_{0j}\sim N(0, \sigma_{1})$ for $j\in\left\{ 1, 2, \ldots, N \right\}$ where N is the number of participants, $u_{0l}$ is the variance at the misinformation post level $l$, $u_{0l}\sim N(0, \sigma_{2})$ for $l\in\left\{ 1, \ldots, 15 \right\}$, and $\varepsilon_{ijl}\sim N(0,\sigma_{ijl}^{2})$ is the variance at the observation level (residual variance).

**Figure S1. Random differences (Arm 2 | Post), ‘reactions’.**

*Posts did not vary in their deviations from the global average effect of the false tag intervention. In other words, posts did not have higher/lower odds of being reacted to than the global average. Posts 1-5 comprised misinformation relating to health; posts 6-10 comprised misinformation relating to politics; and posts 11-15 comprised misinformation relating to finance.*


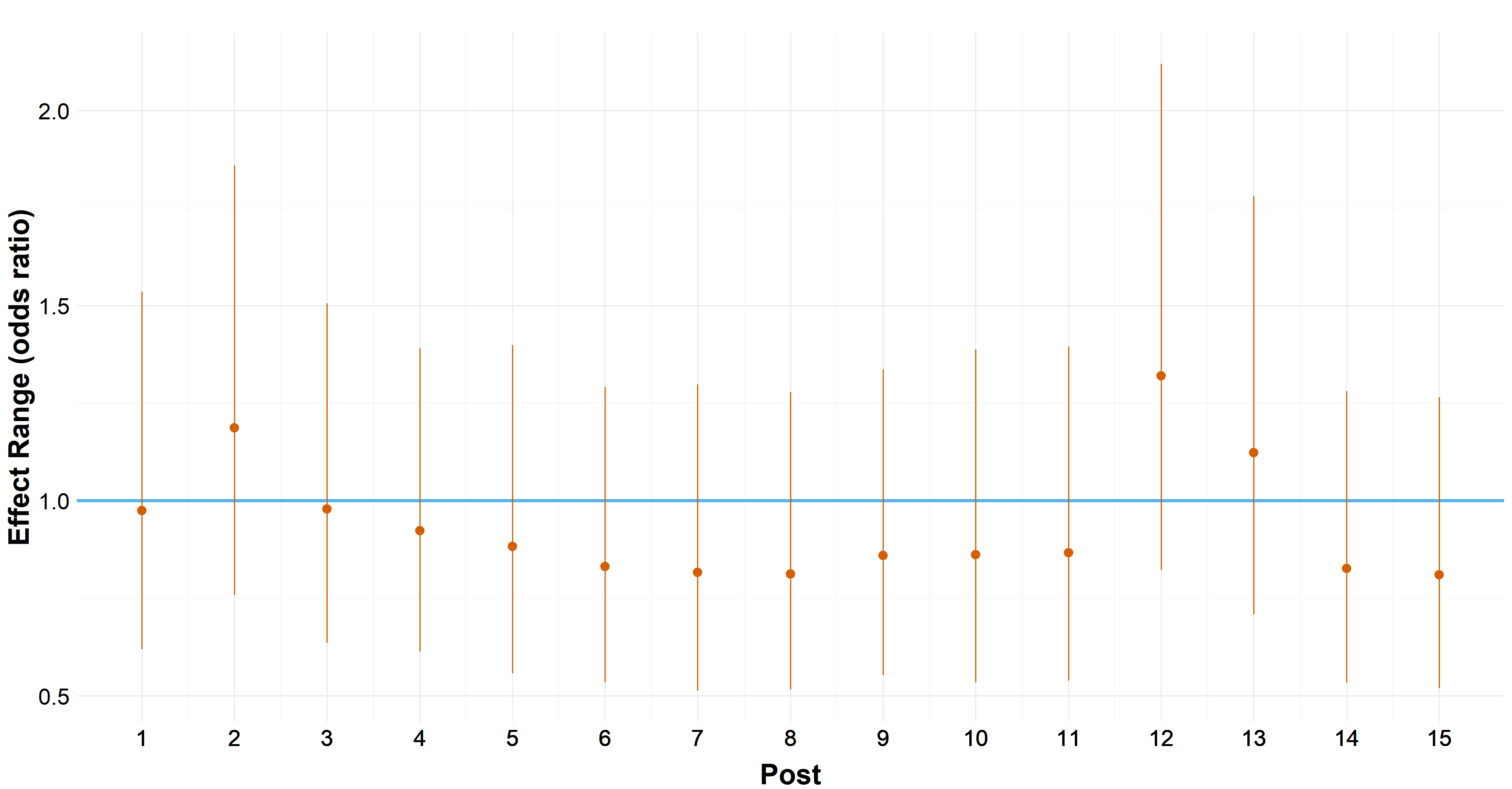

Supplement: Supplementary file 1 — Supplementary Information. [file 41598_2023_32962_MOESM1_ESM.docx]
